# The Mitochondrial Genome Impacts Respiration but Not Fermentation in Interspecific *Saccharomyces* Hybrids

**DOI:** 10.1371/journal.pone.0075121

**Published:** 2013-09-23

**Authors:** Warren Albertin, Telma da Silva, Michel Rigoulet, Benedicte Salin, Isabelle Masneuf-Pomarede, Dominique de Vienne, Delphine Sicard, Marina Bely, Philippe Marullo

**Affiliations:** 1 Univ. de Bordeaux, ISVV, EA 4577, Unité de recherche CEnologie, Villenave d’Ornon, France; 2 Bordeaux Sciences Agro, Gradignan, France; 3 INRA, UMR 0320/UMR 8120 Génétique Végétale, Gif-sur-Yvette, France; 4 CNRS, UMR 5095, Institute of Biochemistry and Genetics of the Cell, Bordeaux, France; 5 Univ. de Bordeaux, IBGC, UMR 5095, Bordeaux, France; 6 Univ Paris-Sud, UMR 0320/UMR 8120 Génétique Végétale, Gif-sur-Yvette, France; 7 BIOLAFFORT, Bordeaux, France; Ben-Gurion University of the Negev, Israel

## Abstract

In eukaryotes, mitochondrial DNA (mtDNA) has high rate of nucleotide substitution leading to different mitochondrial haplotypes called mitotypes. However, the impact of mitochondrial genetic variant on phenotypic variation has been poorly considered in microorganisms because mtDNA encodes very few genes compared to nuclear DNA, and also because mitochondrial inheritance is not uniparental. Here we propose original material to unravel mitotype impact on phenotype: we produced interspecific hybrids between *S. cerevisiae* and *S. uvarum* species, using fully homozygous diploid parental strains. For two different interspecific crosses involving different parental strains, we recovered 10 independent hybrids per cross, and allowed mtDNA fixation after around 80 generations. We developed PCR-based markers for the rapid discrimination of *S. cerevisiae* and *S. uvarum* mitochondrial DNA. For both crosses, we were able to isolate fully isogenic hybrids at the nuclear level, yet possessing either *S. cerevisiae* mtDNA (Sc-mtDNA) or *S. uvarum* mtDNA (Su-mtDNA). Under fermentative conditions, the mitotype has no phenotypic impact on fermentation kinetics and products, which was expected since mtDNA are not necessary for fermentative metabolism. Alternatively, under respiratory conditions, hybrids with Sc-mtDNA have higher population growth performance, associated with higher respiratory rate. Indeed, far from the hypothesis that mtDNA variation is neutral, our work shows that mitochondrial polymorphism can have a strong impact on fitness components and hence on the evolutionary fate of the yeast populations. We hypothesize that under fermentative conditions, hybrids may fix stochastically one or the other mt-DNA, while respiratory environments may increase the probability to fix Sc-mtDNA.

## Introduction

Eukaryotes possess a cytoplasmic organelle called mitochondrion, either fully functional or vestigial [1], [2]. Mitochondria are thought to originate from endosymbiosis between eukaryote’s ancestry and α-proteobacteria. This endosymbiotic event, first proposed by Wallin [3] and popularized by Sagan [4], may have arisen more than two billion years ago [5]. However, nowadays mitochondrial genomes contain far less genes than the genomes of α-proteobacteria [6]. Following endosymbiosis, most of the genes of the endosymbiote were either lost or transferred to the host cell genome during evolution [7]. While mitochondria are complex organelles requiring several hundred proteins to function properly, most of them (>99%) are now the product of nuclear genes. Mitochondrial genomes encode very few genes, between 3 and 96 genes in animals, plants, fungi and protists [7], [8] and the proportion of genes encoded by mtDNA in Eukaryotes usually represents less than 0.5% of the total number of genes.

Mitochondrial gene content varies in a large extent among eukaryotes, with several lineage-specific variations in rates of gene loss. For example, 5 S rRNA is present only in land plants, some green algae, red algae, brown algae and protists [6], implying many independent and repeated losses of the 5 S rRNA gene across eukaryotic evolution. Identically, the number of tRNA genes encoded by mtDNA varies greatly across eukaryotes, ranging from none to around 30 tRNAs genes. In contrast, two major sets of mitochondrial genes are remarkably well conserved, those involved in respiration and in protein synthesis [6].

Unlike nuclear DNA (nuDNA), mtDNA has high rate of nucleotide substitution [9], [10], so that several mitochondrial haplotypes (so-called mitotypes) coexist within species. The analysis of mitochondrial genetic diversity is widely used in population genetics to follow uniparental transmitted markers. However, the importance of mitochondrial genetic variation on phenotypic variation is scarcely considered, firstly because mtDNA encodes very few genes compared to nuDNA, and because mtDNA genetic variation has long been thought to be neutral [11], [12]. In recent years, several studies revisited this longstanding view and showed that mtDNA variation might impact various phenotypic traits [13]. For example, in human, two mtDNA haplotypes were shown to be associated with human survival [14]. Other association studies showed that specific mtDNA mutations in humans are associated with oxygen consumption [15], athletic performance [16], sperm motility [17], Parkinson disease [18], adaptation to diet change and climate [19]. In other animals, cold acclimation was also shown to be associated with mitotypes in the greater white-toothed shrew, *Crocidura russula* [20]. Mitochondrial polymorphism is associated with muscle composition in pig [21] or with resistance to insecticide in an arthropod pest (*Tetranychus urticae*) [22]. Nearly isogenic lines of *Drosophila simulans*, differing for mtDNA, showed important variations for fitness traits (longevity, activity, oxygen consumption, etc) [23]–[25]. In mice, ‘transmitochondrial cybrids’, resulting from the transfer of mitochondria to a mtDNA-less receptor cell line, varied for oxidative phosphorylation performances [26]. In plants and fungi also, cytoplasmic variants are related to fitness traits like in *Silene vulgaris* [27] or in the common button mushroom *Agaricus bisporus* [28].

However, most of these studies were performed at the population level or involved nearly isogenic lines. Indeed, it is very difficult to establish that mtDNA variants are actually associated with phenotype, essentially because nuDNA variations may also be involved. To overcome such difficulties, it is possible to study reciprocal hybrids. Since cytoplasmic organelles are mainly maternally inherited, reciprocal hybridization between two parental lines (♀A x ♂B and ♀B x ♂A) allows the recovery of hybrids displaying identical nuDNA, but differing for organelles’ DNA. Reciprocal hybrids are easily produced for many plants and frequently showed asymmetric phenotypes [29]–[32], but the hybrids differ for both mtDNA and chloroplastic DNA. Thus, assessing unambiguously the role of mtDNA alone requires the use of reciprocal hybrids of non-photosynthetic organisms. This was done for example in *Drosophila* species, where reciprocal hybrids displayed different longevity [33], in reciprocal hybrids of stonechat bird (*Saxicola torquata spp.*) that differ for basal metabolic rate [34], while reciprocal centrarchid fish hybrids had asymmetrical viabilities [35]. However, in most cases, the phenomenon of parental genomic imprinting may be confounded with the effect of mtDNA variability by itself [36].

In this work we took advantage of the particular mitochondrial inheritance of the *Saccharomyces* species [37]. *Saccharomyces* zygotes result from the fusion of two parental cells, each having its own mitochondrial DNA. Thus, in the very first generations after hybridization, hybrids possess both parental mtDNA, which is called heteroplasmy [38]. This heteroplasmic status is only transient and after a few generations (less than 20 divisions), homoplasmic cells harboring only one parental mtDNA are recovered [39]. In some cases, recombination between parental mtDNA may arise [40], yet only one recombined mitotype (homoplasmy) is recovered after a few generations. The transition from heteroplasmy to homoplasmy can be stochastic [41], [42] or non-stochastic [38], [43]. Thus, it is theoretically possible to obtain fully isogenic hybrids resulting from the same cross, but harbouring one or the other of the two parental mtDNA.

In a previous work, Solieri et al. [38] showed that interspecific hybrids between *S. cerevisiae* and *S. uvarum* may have increased respiratory ability when harbouring *S. cerevisiae* mtDNA compared to *S. uvarum* one. However, the synthetic interspecific hybrids tested differed regarding both mtDNA and nuDNA, so that it was difficult to assess whether differences in fermentative and respiratory performances were actually due to mtDNA by itself.

In this work, we produced interspecific hybrids between *S. cerevisiae* and *S. uvarum* species, using fully homozygous diploid parental strains. For two different interspecific crosses involving different parental strains, we recovered 10 independent hybrids per cross, and allowed mtDNA fixation after around 80 generations. For both crosses, we were able to isolate fully isogenic hybrids at the nuclear level, yet possessing either *S. cerevisiae* mtDNA (Sc-mtDNA) or *S. uvarum* mtDNA (Su-mtDNA). These hybrids were used to test the phenotypic impact of mitochondrial inheritance under respiratory conditions. In addition, even though it has long been suggested that mtDNA do not play any role in fermentation, indirect evidences suggested that actually they could [44]. Accordingly Sc-mtDNA and Su-mtDNA hybrids were also compared under fermentative conditions.

## Materials and Methods

### Yeast Strains and Culture Conditions

Eleven strains of *Saccharomyces cerevisiae* and four strains of *S. uvarum* were selected (Table 1). Monosporic clones were isolated from all these strains using a micromanipulator (Singer MSM Manual; Singer Instrument, Somerset, United Kingdom). All strains but Alcotec 24 and NRRL-Y-7327 were homothallic (*HO*/*HO*), so that the monosporic derivates were fully homozygous diploid. For Alcotec 24 and NRRL-Y-7327 (*ho/ho*), the isolated haploid meiospore were diploidized via transient expression of the HO endonuclease (see Albertin et al., 2009 [45]). These fully homozygous diploid strains, called W1–W2, D1–D2, B1–B2, E1–E5 for *S. cerevisiae* and U1–U4 for *S. uvarum* were used for subsequent analysis of the genetic diversity of mitochondrial DNA and for interspecific hybrid construction.

**Table 1:** Characteristics of *Saccharomyces cerevisiae* and *S. uvarum* strains used.

Species	Strain	Genotype	Ploidy	Collection/supplier^a^	Origin	Reference
*S. cerevisiae*	YSP128	*HO/HO*	diploid	SGRP	Forest Oak exudate,Pennsylvania, USA	Liti et al., 2009 [60]
*S. cerevisiae*	UWOPS83-787.3	*HO/HO*	diploid	SGRP	Fruit *Opuntia stricta*, Bahamas	Liti et al., 2009 [60]
*S. cerevisiae*	Alcotec 24	*ho/ho*	diploid	Hambleton Bard	Distillery, UK	Albertin et al., 2011 [46]
*S. cerevisiae*	CLIB-294	*HO/HO*	diploid	CIRM-Levures	Distillery, Cognac, France	Albertin et al., 2011 [46]
*S. cerevisiae*	CLIB-328	*HO/HO*	diploid	CIRM-Levures	Enology, UK	Albertin et al., 2011 [46]
*S. cerevisiae*	CLIB-382	*HO/HO*	diploid	CIRM-Levures	Brewery, Japan	Albertin et al., 2011 [46]
*S. cerevisiae*	VL1	*HO/HO*	diploid	Laffort Œnologie	Enology, Bordeaux, France	Marullo et al., 2006 [88]
*S. cerevisiae*	F10	*HO/HO*	diploid	Laffort Œnologie	Enology, Bordeaux, France	Marullo et al., 2009 [89]
*S. cerevisiae*	VL3c	*HO/HO*	diploid	Laffort Œnologie	Enology, Bordeaux, France	Marullo et al., 2004 [90]
*S. cerevisiae*	BO213	*HO/HO*	diploid	Laffort Œnologie	Enology, Bordeaux, France	Marullo et al., 2006 [88]
*S. cerevisiae*	NRRL-Y-7327	*ho/ho*	diploid	NRRL	Brewery, Tibet	Albertin et al., 2009 [45]
*S. uvarum*	PM12	*HO/HO*	diploid	ISVV	Grape must, Jurançon, France	Naumov et al., 2000 [91]
*S. uvarum*	PJP3	*HO/HO*	diploid	ISVV	Grape must, Sancerre, France	Naumov et al., 2000 [91]
*S. uvarum*	Br6.2	*HO/HO*	diploid	ADRIA Normandie	Cider fermentation, Normandie, France	
*S. uvarum*	RC4-15	*HO/HO*	diploid	ISVV	Grape must, Alsace, France	Demuyter et al., 2004 [92]
*S. cerevisiae*	W1	monosporic clone ofYSP128, HO/HO	diploid	ISVV		Blein et al., 2013 [62]
*S. cerevisiae*	W2	monosporic clone ofUWOPS83-787.3, HO/HO	diploid	ISVV		this work
*S. cerevisiae*	D2	monosporic clone ofAlcotec 24, ho/ho	diploid	ISVV		Albertin et al., 2011 [46]
*S. cerevisiae*	D1	monosporic clone ofCLIB-294, HO/HO	diploid	ISVV		Albertin et al., 2011 [46]
*S. cerevisiae*	E1	monosporic clone ofCLIB-328, HO/HO	diploid	ISVV		Albertin et al., 2011 [46]
*S. cerevisiae*	B1	monosporic clone ofCLIB-382, HO/HO	diploid	ISVV		Albertin et al., 2011 [46]
*S. cerevisiae*	E3	monosporic clone ofVL1, HO/HO	diploid	ISVV		Albertin et al., 2011 [46]
*S. cerevisiae*	E4	monosporic clone ofF10, HO/HO	diploid	ISVV		Albertin et al., 2011 [46]
*S. cerevisiae*	E5	monosporic clone ofVL3c, HO/HO	diploid	ISVV		Blein et al., 2013 [62]
*S. cerevisiae*	E2	monosporic clone ofSB, HO/HO	diploid	ISVV		Marullo et al., 2009 [89]
*S. cerevisiae*	B2	monosporic clone ofNRRL-Y-7327, ho/ho	diploid	ISVV		Blein et al., 2013 [62]
*S. uvarum*	U1	monosporic clone ofPM12, HO/HO	diploid	ISVV		Blein et al., 2013 [62]
*S. uvarum*	U2	monosporic clone ofPJP3, HO/HO	diploid	ISVV		Blein et al., 2013 [62]
*S. uvarum*	U3	*monosporic clone of* *Br6.2, HO/HO*	diploid	ISVV		Blein et al., 2013 [62]
*S. uvarum*	U4	*monosporic clone of* *RC4-15, HO/HO*	diploid	ISVV		this work
*S. cerevisiae*	D2-3A-HYG	*ho::hygR, MATα*	haploid	ISVV		this work
*S. cerevisiae*	W1-NAT-1B	*ho::natR, MATa*	haploid	ISVV		this work
*S. uvarum*	U2-KAN-3B	*Suho::kanR, MATα*	haploid	ISVV		this work
*S. uvarum*	U3-KAN-3A	*Suho::kanR, MATa*	haploid	ISVV		this work

a Laffort CEnologie: http://www.laffort.com; CIRM-Levures (Centre International de Ressources Microbiennes): http://www.inra.fr/internet/Produits/cirmlevures; NRRL (Northern Regional Research Laboratory, now Agricultural Research Service Culture Collection): http://nrrl.ncaur.usda.gov; Hambleton Bard: http://www.hambletonbard.com; ISVV (Institut Scientifique de la Vigne et du Vin): http://www.oenologie.u-bordeaux2.fr/; ADRIA Normandie: http://www.adria-normandie.com; SGRP (Saccharomyces Genome Resequencing Project): http://www.sanger.ac.uk/research/projects/genomeinformatics/sgrp.html.

All strains were usually grown at 24°C in YPD medium containing 1% yeast extract (Difco Laboratories, Detroit, MI), 1% Bacto peptone (Difco), and 6% glucose, supplemented or not with 2% agar. When necessary, antibiotic concentration was as followed: 100 µg/mL for G418 (Sigma, France), 300 µg/mL for hygromycin B (Sigma, France), and 100 µg/mL for nourseothricin (Sigma, France).

For a quick assessment of respiratory-ability, cells were plated on YPGly medium, containing glycerol as unique source of carbon: 1% yeast extract (w/v, Difco Laboratories, Detroit, MI), 1% Bacto peptone (w/v, Difco), 2% (v/v) glycerol and 2% (w/v) agar.

### Mitochondrial DNA Sequence

Genomic DNA extraction were performed as described by Albertin *et al* [46] or by using FTA® CloneSaver ™ Card (Whatman®BioScience, USA). Three mitochondrial loci, *COX3, COX2* and *ATP6,* were sequenced in 11 *S. cerevisiae* and 4 *S. uvarum* fully homozygous strains. An additional locus *VAR1* was sequenced only for *S. cerevisiae* strains. Both strands of PCR products were sequenced using Sanger method (GATC biotech, Germany). The sequences were aligned with ClustalW using the BioEDIT program [47]. Aligned fragments were deposited in EMBL (accession numbers HF951715–HF951770). The genetic distance between sequences (number of differences per base) was estimated using MEGA 5 software [48]. Phylogenic trees were build using the Neighbor-Joining method [49] with bootstrap implementation (500 iterations).

### Mitochondrial Genotyping

The three loci *COX2*, *COX3* and *ATP6* were used to design degenerated primers able to amplify in a single PCR reaction the *S cerevisiae* and *S uvarum* alleles. The *COX2* primers used were previously described by Belloch *et al.* [50] and required the digestion of PCR fragment by the endonuclease *Sfc*I. The *ATP6* and *COX3* primers allow differentiating *S. uvarum* and *S. cerevisiae* by the PCR product length (Table S1). An additional locus, *VAR1* can be used to discriminate *Saccharomyces cerevisiae* strains by an RFLP approach (Table S1). The PCR reactions were carried out with 2–6 ng of genomic DNA extract as template, 1X Taq-&GO master mix for PCR (Qbiogene), in 20 µL final volume. PCR fragment sizes were analyzed by capillary electrophoresis with a multi NA apparatus (Shimatzu, Germany) using the 1000 pb gel kit.

### Hybrid Construction

In order to produce interspecific hybrids, two diploid parental strains per species (W1 and D2 for *S. cerevisiae*, U2 and U3 for *S. uvarum*) were transformed with a cassette containing the *HO* allele disrupted by a gene resistance to either G418 (*ho::KanR*), hygromycin B (*ho::HygR*) or nourseothricin (*ho::NatR*). For *S. cerevisiae* strains, the *ho::KanR*, *ho::HygR* and *ho::NatR* cassettes were respectively amplified by PCR using the following primers p25: TGGTTTACGAAATGATCCACG, p26: AAATCGAAGACCCATCTGCT and the genomic DNA of the strains BY4741 (Euroscarf, Franckfurt, Germany), RG1 and RG13 (kindly given by Professor Richard Gardner, Auckland, New Zealand). For *S. uvarum* strains, the *Suho::KanR* cassette containing the KanMX4 coding sequence (1506 pb) flanked on 5′ and 3′ by 500 pb flanking- sequence of *S. uvarum HO* gene was synthesized by Genscript and cloned in the pUC57 vector. This cassette was then amplified by PCR using the primers p599 TACCACGAAAAACTGATGTAATGG and p600 CTTTATCTGACGCTATGGCCG. For all ho-disruption cassettes amplification, the PCR mix contained 100–600 ng of DNA template, 0.1 mM of each primer, 1X Taq-&GO master mix for PCR (Qbiogene), in 100 µl final volume. The PCR reaction was as followed: 3 minutes at 94°C, followed by 35 cycles –30 seconds at 94°C, 30 seconds at 54°C or 55°C (for *S. cerevisiae* and *S. uvarum* cassettes respectively), 3 minutes at 72°C – and a final elongation step of 5 minutes at 72°C.

Strains were transformed using the lithium acetate protocol described by Gietz and Schiestl [51] for all *S. cerevisiae* strains and for U4, and alternatively using the frozen yeast TRAFO protocol [52] for U1, U2 and U3. After transformation, monosporic clones were isolated, and the mating-type (*MATa* or *MATα*) of antibiotic-resistant clones was determined using testers of well-known mating-type. Strain transformation allowed (i) conversion to heterothallism for the homothallic strains (all but B2 and D2, see Table 1) and (ii) antibiotic resistance allowing easy hybrid production.

For DU23 hybrids, the parental strains D2-3A-HYG (*MATα*) and U3-KAN-3A (*MATa*) were pulled in contact two to four hours in YPD medium at room temperature, and then plated on YPD-agar with G418 and hygromycinB. The same procedure was applied for WU12 hybrids whose parental strains were W1-NAT-1B (*MATa*) and U2-KAN-3B (*MATα*) and were thus selected on YPD-agar added with G418 and nourseothricin. Ten independent hybrids per cross were recovered. Recurrent cultures on YPD-agar (24°C), each from one colony, which corresponded to ∼80 generations, were made in order to allow mitochondrial fixation (homoplasmy) and to assess hybrids chromosomal stability through multiple generations.

### Hybrid Characterization

Karyotype analysis of the hybrids and their corresponding progenitors was carried out using pulse-field gel electrophoresis (PFGE). Briefly, chromosomal DNA was prepared from overnight cultures in agarose plugs as described by Bellis et al. [53]. Chromosomes were separated with a CHEF DRII apparatus (Bio-Rad, Richmond, CA, USA) on a 1% agarose gel (Qbiogene, Carlsbad, CA, USA) and using TBE as running buffer. Electrophoresis was carried out at 200 V and 10°C for 16 h with a switching time of 60 ms, and then for 10 h with a switching time of 105 ms. DNA was bound by bromide ethidium staining (30 minutes).

In addition to PFGE, hybrids were characterized by PCR ribotyping (5.8S-ITS rDNA amplification followed by *Hae*III restriction) allowing discrimination between *S. cerevisiae* and *S. uvarum* strains [38], [54], [55].

### Fermentation Assays

White grape must was obtained from Sauvignon grapes, harvested in vineyards in Bordeaux area (2009 vintage). Tartaric acid precipitation was stabilized and turbidity was adjusted to 100 NTU (Nephelometric Turbidity Unit) before long storage at –20°C. Sugar concentration was 188 g L^×1^, and the indigenous yeast population, estimated by YPD-plate counting after must thawing, was low, *i.e.* less than 20 CFU (colony-forming unit) *per* mL.

Pre-cultures were run in half-diluted must filtered through a 0.45 µm nitrate-cellulose membrane, during 24 h, at 24°C with orbital agitation (150 rpm). Population size was measured using a flow cytometer (see below). Sauvignon must was inoculated at 10^6^ viable cells *per* mL. Fermentation triplicates were run in closed 125 mL glass-reactors, locked to maintain anaerobiosis, with permanent stirring (300 rpm) at 18°C. The CO_2_ released was allowed by a needle and was determined by measurement of glass-reactor weight loss regularly and the CO_2_max was calculated as the maximal CO_2_ released in g L^–1^. The fermentation kinetics data were fitted with logistic model allowing the calculation of several kinetics parameters: *lag phase time* (h) was the time between inoculation and the beginning of CO_2_ release. *AF time* (h) was the time to complete alcoholic fermentation (without lag-phase). *Vmax* was the maximal rate of CO_2_ release in g L^–1^ h^–1^.

At the end of the alcoholic fermentation, ethanol concentration (percent volume) was determined by infrared reflectance (Infra-Analyzer 450; Technicon, Plaisir, France), acetic acid production (g L^−1^) were measured by colorimetry (*A*460) in continuous flux (Sanimat, Montauban, France) and both residual D-glucose and D-fructose (g L^–1^) were quantified using an enzymatic method (Kit D glucose/D fructose Boehringer, Germany) in the supernatant.

External glycerol (g L-1) was assayed by the enzymatic method (Boehringer kits 10 148 270 035, R-Biopharm, Darmstadt, Germany).

### Cell Growth Conditions for Respiratory Assays

Respiratory growth was assessed on YPEG medium containing 1% yeast extract (w/v, Difco Laboratories, Detroit, MI), 1% Bacto peptone (w/v, Difco, Detroit, MI), 3% ethanol (v/v) and 3% glycerol (v/v). Pre-cultures were run in half-diluted YPEG medium during 24 h, at 28°C with orbital agitation (150 rpm). Population size was measured using flow cytometry (see below) to inoculate YPEG at 10^6^ viable cells *per* mL. Triplicates were run in 200 mL Erlenmeyers containing 50 mL YPEG medium, with high permanent stirring (900 rpm) to favour oxygenation at 28°C.

### Population Dynamics Using Flow Cytometry

Regularly, cells were sampled and population size was estimated using a flow cytometer (Quanta SC MPL, Beckman Coulter, France), equipped with a 488 nm laser and a 670 nm long-pass filter, at 22 mW. Samples were diluted in McIlvaine buffer pH4 (0.1 M citric acid, 0.2 M sodium phosphate dibasic) added with propidium iodide (0.3% v/v) in order to stain dead cells (red fluorescence measure in FL3 channel). The experimental points were fitted with a logistic model [46] that allowed estimation of the carrying capacity (maximum population size, *K*, cells per mL) and the intrinsic growth rate *r* (number of divisions *per* hour).

### Oxygen Consumption Assays

WU12-8 (Sc-mtDNA) and WU12-1 (Su-mtDNA) were grown aerobically in YPEG liquid medium, at 28°C. During exponential phase, the oxygen consumption was measured polarographically at 28°C using a Clark oxygen electrode in a 1-mL thermostatically controlled chamber. Distinct respiratory rates were considered: spontaneous respiratory rate (*JO_2_*, which is oxygen uptake during growth conditions), uncoupled respiratory rate (*JO_2_max*), which is measured in the presence of 1 µM of the protonophoric uncoupler CCCP (carbonyl cyanide m-chlorophenylhydrazone, Sigma, France) and is an indication of the maximal respiratory rate achieved by the cells [56], and finally none-phosphorating respiratory rate (*basal JO_2_*) which is the residual respiratory rate measured when ATPsynthase is inhibited in presence of 200 µM of TET (Tri Ethyl Tin chloride, Alfa Aesar, USA). The ATP respiratory rate (*JO_2_ATP*) was calculated as the difference between spontaneous *JO_2_* and *basal JO_2_*, and the percentage of spontaneous respiration due to ATPase activity was estimated (*JO_2_ATP/JO_2_*). All respiratory rates were determined from the slope of a plot of O_2_ concentration versus time and were expressed as nmol O_2_/min/10^e6^ cells, population size being measured by flow cytometry. Four measures of all three respiratory rates were performed during exponential growth, and the experiment was performed in duplicate.

### Cytochrome Content Determination

The cellular content of *c+c1*, *b*, and *a+a3* hemes was calculated as described by Dejean et al. [57], taking into account the respective molar extinction coefficient values and the reduced minus oxidized spectra recorded using a dual beam spectrophotometer (Aminco DW2000). Two to four measures were made, and cytochrome content was expressed in pmol/mg dry weight of cells.

### Electronic Microscopy

Yeast pellets (after YPEG overnight growth) were placed on the surface of a copper EM grid (400 mesh) that had been coated with formvar. Each grid was very quickly submersed in liquid propane pre-cooled and held at −180°C by liquid nitrogen. The loops were then transferred in a pre-cooled solution of 4% osmium tetroxide in dry acetone in a 1.8 ml polypropylene vial at −82°C for 72 h (substitution), warmed gradually to room temperature, followed by three washes in dry acetone. Specimens were stained for 1 h in 1% uranyl acetate in acetone at 4°C, blackroom (epoxy resin Fluka). Ultrathin sections were contrasted with lead citrate. Specimens were observed with a HITACHI 7650 (80 kV) electron microscope (PIE, BIC, Bordeaux Segalen University).

### Statistical Analysis

Within each cross (WU12 and DU23), the variation of each trait was investigated using the lm function (R program), through the following model of ANOVA:
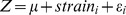
where Z is the variable, *strain* is the strain effect (*i* = 1, 2, 3, 4) and *ε* is the residual error. Within each cross, the four strains corresponded to two independent strains with Sc-mtDNA and two independent strains with Su-mtDNA. Since several traits were tested, *P* values were adjusted for multiple testing using Benjamini-Hochberg methods by means of R’s language, version 2.14.1 [58]. For each variable, the homogeneity of the variance was assessed using a Levene test by means of R’s *car* package version 2.14.1 [58], as well as the normality of residual distribution using a Shapiro test [58]. Duncan’s multiple comparison was used to determine which means differ significantly (Duncan’s multiple comparison, *p*<0.05).

### *In silico* Competition between Mitotypes

Modeling population growth was made using the kinetics parameters calculated under respiratory conditions (YPEG medium) using a logistic model: *K* = 3.63.10^8^ cells per mL for both WU12 Sc-mtDNA and Su-mtDNA, *r* = 0.222 and 0.196 division per hour for WU12 Sc-mtDNA and Su-mtDNA respectively; *K* = 3.29. 10^8^ cells per mL for both DU23 Sc-mtDNA and Su-mtDNA, *r* = 0.207 and 0.176 division per hour for DU23 Sc-mtDNA and Su-mtDNA respectively. The initial mixed population was of 10^6^ cells per mL (ratio 1∶1 Sc-mtDNA:Su-mtDNA). When the maximal population size was reached (*K*), a new *in silico* culture was inoculated at 10^6^ cells per mL, using the ratio of mitotypes (Sc-mtDNA:Su-mtDNA) calculated at the end of the preceding culture.

## Results

### Mitochondrial Sequence Analysis in *S. cerevisiae* and *S. uvarum*

**Figure 1:**
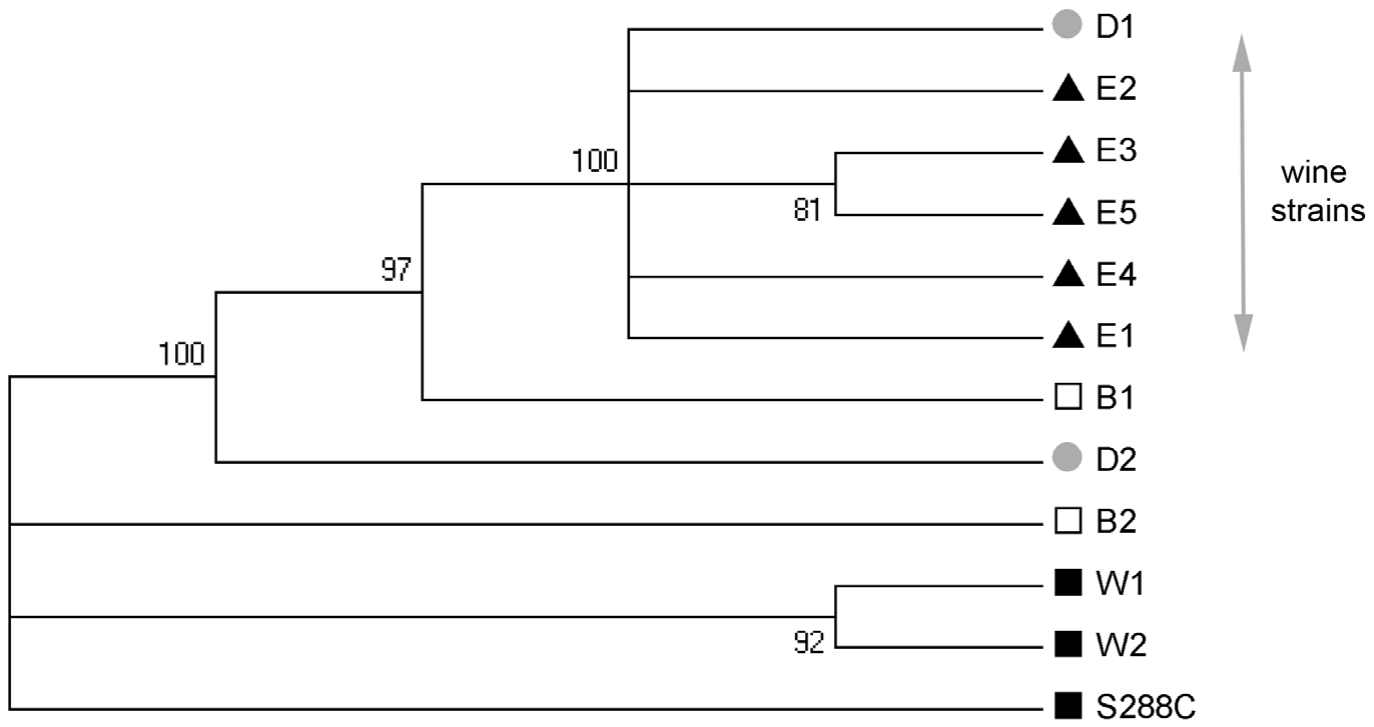
Evolutionary relationships *of Saccharomyces cerevisiae* strains for mtDNA. The phylogenic tree was inferred by using the Maximum Likelihood method based on the Tamura-Nei model with bootstrapping (500 iterations). Branches corresponding to partitions reproduced in less than 80% bootstrap replicates are collapsed. The analysis involved 12 nucleotide sequences representing the concatenation of 4 mitochondrial loci (*COX2*, *COX3*, *VAR1* and *ATP6*). All positions containing gaps and missing data were eliminated. There were a total of 2719 positions in the final dataset. Evolutionary analyses were conducted in MEGA5. Label describes the origin of the strains: natural isolates▪, distillery•, brewing□, wine▴.

In order to develop polymorphic mitochondrial markers for both *S. cerevisiae* and *S. uvarum* species, we sequenced three mitochondrial genes (*COX3, COX2 and ATP6)* for 11 *S. cerevisiae* and 4 *S. uvarum* strains. An additional loci *VAR1* was sequenced only for *S. cerevisiae* strains. To maximize the chance to find polymorphism, intergenic segments were amplified from flanking coding regions. This dataset allows a first study of the intra-specific variability of mtDNA within natural populations (Table 2). For *S. cerevisiae*, sequence alignments of *COX2*, *ATP6*, *COX3,* and *VAR1* were performed for 12 strains, including the reference strain S288C. Depending on the gene, we identified 5 to 11 allelic forms. The genetic polymorphism varied greatly depending on the locus and the strain with an average of 2.33% nucleotide difference within the 12 strains. The *COX2* and *VAR1* coding sequences display low polymorphism (0.34% and 0.43% nucleotide difference). By contrast, the promoters of *ATP6* and *COX3* promoter harbored more nucleotide polymorphism between strains (9.75% and 0.66% nucleotide difference, respectively). The promoter region of *ATP6* was found to be particularly polymorphic due to the insertion of two CG clusters at different position defining two groups of strains. A multi-locus analysis was carried out concatenating these sequences (2650 positions). Wine yeasts were grouped together as illustrated by the phylogenic three presented in Figure 1, which is congruent with previous work studying nuclear DNA polymorphism [45], [59], [60].

**Table 2:** Genetic diversity of *COX2, COX3*, *ATP6* and *VAR1* mtDNA loci.

Locus	Species(# strains)	Alignmentsize	Allelesnumber	Nucleotidedifference range^c^	Description	EMBLaccess
*COX2*	*S cerevisiae* (12) a	527	5	0–4	*COX2* coding sequence	HF951745-48
	*S uvarum* (4) b	561	2	0		HF951749-60
*COX3*	*S cerevisiae* (12)	630–749	7	0–78	*COX3* promoter	HF951734-44
	*S uvarum* (4)	704-507	3	0–6		HF951730-33
*ATP6*	*S cerevisiae* (12)	692–743	11	12–366	*ATP6* promoter	HF951719-29
	*S uvarum* (4)	450–480	4	0–7		HF951715-18
*VAR1*	*S cerevisiae* (12)	971–1068	7	0–145	*VAR1* coding sequence	HF951760-70
	*S uvarum*	ND	ND	ND		

a For *S. cerevisiae*, 12 sequences (11 strains+reference strain) were analyzed.

b For *S. uvarum*, 4 sequences were analyzed, the sequence of the strain PM12 was used as reference.

c Number of base differences per sequence respect to the reference. Results are based on the pairwise analysis conducted in MEGA5; all positions containing alignment gaps and missing data were eliminated only in pairwise sequence comparisons.

For *Saccharomyces uvarum*, there is no published mitochondrial genome. So we used *S. pastorianus* mtDNA genome as reference: *S. pastorianus* is an allotetraploid whose progenitors are *S. cerevisiae* and *S. eubayanus*, a newly-described species phylogenetically closed to *S. uvarum*. *S. pastorianus* inherited the mitochondrial DNA from S. *eubayanus* [61]. Regarding the three loci analyzed (*COX2*, *COX3* and *ATP6*), the *S. eubayanus* mtDNA sequence is divergent from the four *S. uvarum* sequences with an average of 8.8% nucleotide difference for 1454 positions, while within *S. uvarum* few allelic variations were detected (0.30% nucleotide difference). Such a low genetic variability within *S. uvarum* in comparison to *S. cerevisiae* is consistent with the results of a recent multilocus genotyping experiment carried out on six nuclear genes [62].

### Development of Co-dominant Mitochondrial Markers for *S. cerevisiae* and *S. uvarum* Species

**Figure 2:**
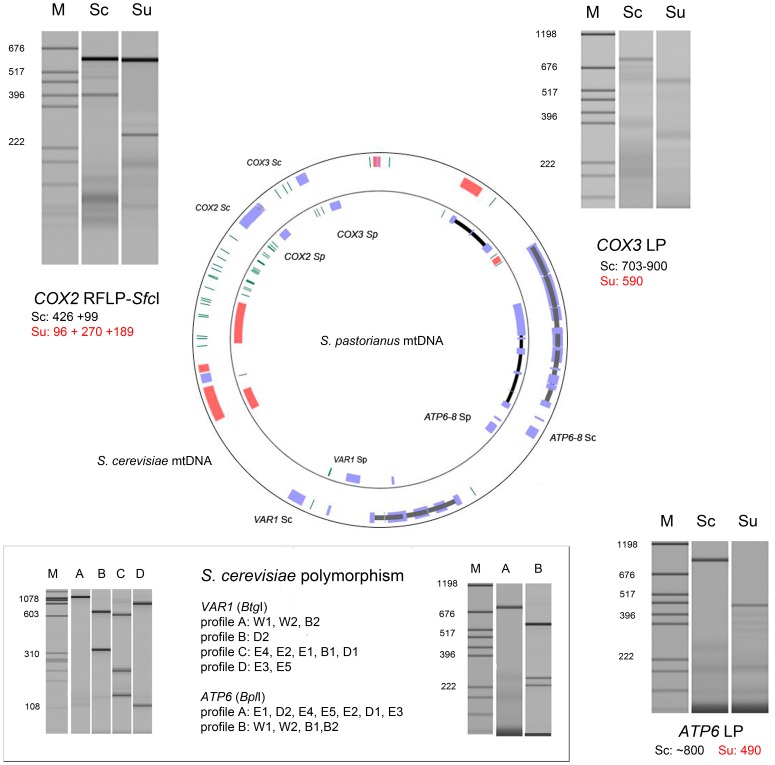
Molecular markers for typing intra and interspecific variability of mtDNA in *S. cerevisiae* and *S. uvarum* species. Three interspecific markers (*S. cerevisiae* vs. *S. uvarum*) and two intra *S. cerevisiae* markers were developed using PCR and enzymatic restriction. The interspecific markers *ATP6* and *COX3* allowed the rapid identification of mitotypes by length polymorphism after PCR. The *COX2* marker required the digestion of PCR fragments by the enzyme *Sfc*I to discriminate the two species mitotypes. For the identification of mtDNA within *S. cerevisiae* strains the *ATP6* and *VAR1* PCR fragments were digested with the restriction enzymes *Bpl*I and *Bgt*I, respectively. Combining both markers, five mitotypes could be identified.

To have a readily and economic mtDNA genotyping, co-dominant mitochondrial markers were developed using either variation in length PCR-amplicon (PCR-LP) or PCR followed by RFLP. Although numerous nucleotide polymorphisms were found by sequencing, a relative few number of restriction sites were observed. For inter-specific discrimination, three markers were developed (*COX3*, *COX2* and *ATP6*) that allowed a clear discrimination between *S. cerevisiae* and *S. uvarum* (Figure 2). In addition, *ATP6* and *VAR1* loci displayed intra-specific polymorphism within *S. cerevisiae* species when the PCR product was digested with *Bpl*I and *Btg*I respectively. When combined together, those loci allowed differentiating five of the 11 mtDNA of the *S. cerevisiae* strains analyzed (Figure 2). By contrast, the very low polymorphism of *S. uvarum* species prevents the use of these mtDNA markers to discriminate *S. uvarum* strains (Table S1).

### Interspecific Hybrid Construction and Characterization

**Figure 3:**
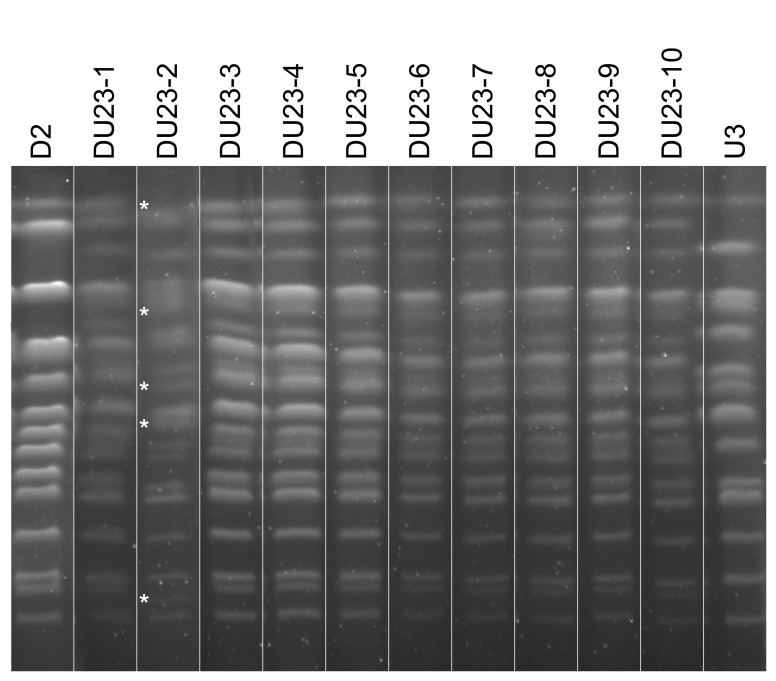
Karyotype analysis of the *S. cerevisiae* strain D2, *S. uvarum* strain U3 and their interspecific hybrids DU23. Pulse field gel electrophoresis was performed on 10 independent DU23 interspecific hybrids. Stars indicate absent parental chromosomes or chromosomes of unexpected size for DU23-2 interspecific hybrid.

Interspecific hybridization between *S. cerevisiae* and *S. uvarum* was performed, allowing us to get the hybrids DU23 (D2×U3) and WU12 (W1×U2). For each cross, ten independent hybrids were isolated and confirmed by amplification of the rDNA *NTS2* region followed by *Hae*III restriction [63]. Recurrent cultures were then made, corresponding to 80 generations. Pulse-field gel electrophoreses were run to determine whether the hybrids actually possessed both parental chromosome sets. All 20 hybrids displayed additive karyotype, except DU23-2 (Figure 3) that presented large chromosomal rearrangements with additional and missing parental chromosome bands. This result indicated that inter-specific hybridization was relatively stable at the chromosomal level, even after 80 generations.

Mitochondrial inheritance was then assessed for these 20 interspecific hybrids to determine whether the different hybrids had recovered Sc-mtDNA or Su-mtDNA. The mtDNA was genotyped after 20 and 80 generations (Table S2). Depending on the interspecific cross, the results varied: after 20 generations, only one case of heteroplasmy was detected among 10 independent WU12 hybrids, and after 80 generations all 10 hybrids had fixed either Sc-mtDNA (7/10 hybrids) or Su-mtDNA (3/10 hybrids). All these interspecific hybrids were able to grow on YPGly petri plate, containing glycerol as carbon source, indicating efficient respiration metabolism. By contrast, for DU23 background, two heteroplasmic hybrid strains were observed after 20 generations, as well as three hybrids with mtDNA recombination, most of them being respiratory-defective (unable to grow on YPGly petri plate). After 80 generations, four inter-specific hybrids displayed partial or complete mtDNA loss, associated with inability to grow on YPGly petri plate. Two hybrids with mtDNA recombination were observed, of which only one was able to respire. The four remaining inter-specific hybrids were homoplasmic, two of them with Sc-mtDNA, and two with Su-mtDNA.

### Hybrids with Sc-mtDNA have Higher Growth Performance Under Respiratory Conditions

**Figure 4:**
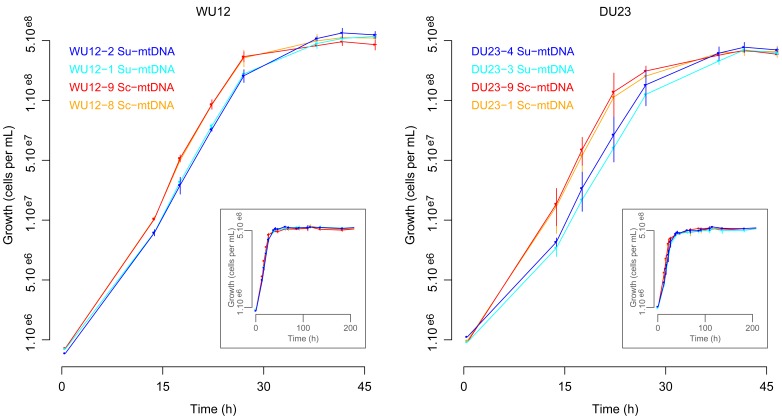
Growth dynamics under respiratory conditions for WU12 and DU23 interspecific hybrids. Population growth was assessed on YPEG medium, using flow cytometry. For each strain, triplicates were made and error bars show standard deviations. The growth kinetics are represented in small captions, while large captions focus on the first part of growth dynamics.

The possibility to obtain readily numerous inter-specific hybrids by antibiotic selection and the development of molecular test for assessing mitochondrial inheritance pave the way to investigate the phenotypic impact of mitochondrial inheritance in an isogenic context. This unique genetic material allows evaluating the impact of natural genetic variations of mtDNA on the fitness of inter-specific hybrids. For each interspecific cross (WU12 and DU23), we chose two independent homoplasmic hybrids with either Sc-mtDNA (WU12-8, WU12-9, DU23-1 and DU23-9) or Su-mtDNA (WU12-1, WU12-2, DU23-3 and DU23-4). These hybrids are thus fully identical at the nuclear level and differ only for mitochondrial DNA, allowing reliable study of the impact of mtDNA inheritance alone on phenotype. As the foremost function of mitochondria in yeast is glucose oxidation through cellular respiration, we first analyzed cell growth under respiratory conditions. The interspecific hybrids were grown in YPEG medium associated with strong permanent stirring, and population was followed by flow cytometry analysis. For both crosses, interspecific hybrids having Su-mtDNA (WU12-1, WU12-2, DU23-3 and DU23-4) had apparent lower population size until the carrying capacity (maximal population size) was reached (Figure 4). Growth kinetics were fitted on logistic function to determine the lag phase time, the maximal population size *K*, and the intrinsic growth rate *r*. Variance analysis (ANOVA) revealed that interspecific hybrids reached similar maximal population size within each cross, indicating that mtDNA inheritance had no impact on final carrying capacity in interspecific hybrids (Table 3). By contrast, lag phase time and intrinsic growth rate were strongly affected: for WU12 cross, hybrids with Su-mtDNA had increase lag phase time (15.4 and 16.1 hours for WU12-1 and WU12-2 respectively) than hybrids with Sc-mtDNA (13.2 and 13.8 hours for WU12-8 and WU12-9, respectively). In addition, Su-mtDNA hybrids showed lower intrinsic growth rate than hybrids with Sc-mtDNA (0.201 and 0.191 division per hour for WU12-1 and WU12-2 respectively, compared to 0.224 and 0.221 division per hour for WU12-8 and WU12-9, respectively). The same features were observed for DU23 cross, with Su-mtDNA hybrids having higher lag phase time (around 16.6 hours) and lower growth rate (around 0.175) compared to Sc-mtDNA hybrids (14 hours of lag phase and 0.207 division per hour). In both interspecific crosses, Su-mtDNA inheritance was associated with delayed and slower growth compared to Sc-mtDNA.

**Table 3:** Results of the ANOVAs: F values and Mean values for respiration parameters.

	WU12 interspecific cross						DU23 interspecific cross					
	ANOVA		Mean value +/− SD (Duncan’s class)				ANOVA		Mean value +/− SD (Duncan’s class)			
Parameters	Fvalue	df	WU12-1 Su	WU12-2 Su	WU12-8 Sc	WU12-9 Sc	Fvalue	Df	DU23-1 Sc	DU23-3 Su	DU23-4 Su	DU23-9 Sc
*K*	3,34	3	3.96e+08+/−1.9e+07	3.91e+08+/−2.8e+07	3.51e+08+/−1.4e+07	3.75e+08+/−1.2e+07	1,92	3	3.44e+08+/−5e+06	3.49e+08+/−2.5e+07	3.16e+08+/−2.6e+07	3.13e+08+/−2.9e+07
*R*	9,38**	3	0.201+/−0.006(a)	0.191+/−0.007(a)	0.224+/−0.008(b)	0.221+/−0.013(b)	7,85*	3	0.204+/−0.01(b)	0.178+/−0.009a	0.173+/−0.002(a)	0.211+/−0.019(b)
*lag-phase*	9,22**	3	15.38+/−0.01(b)	16.07+/−0.01(b)	13.25+/−0.01(a)	13.79+/−0.01(a)	11,45*	3	14.52+/−0.01(a)	16.65+/−0.01(b)	16.57+/−0(b)	13.59+/−0.02(a)
*JO_2_*	181,55***	1	1.07+/−0.13(a)	ND	3.03+/−0.39(b)	ND	ND	ND	ND	ND	ND	ND
*JO_2_max*	66,10***	1	1.67+/−0.26(a)	ND	3.67+/−0.64(b)	ND	ND	ND	ND	ND	ND	ND
*JO_2_ATP*	155,63***	1	0.78+/−0.15(a)	ND	2.31+/−0.32(b)	ND	ND	ND	ND	ND	ND	ND
*basal JO_2_*	35,87***	1	0.29+/−0.06(a)	ND	0.72+/−0.19(b)	ND	ND	ND	ND	ND	ND	ND
*JO_2_ATP/JO_2_*	1,37	1	0.73+/−0.07	ND	0.76+/−0.05	ND	ND	ND	ND	ND	ND	ND
*c+c1*	133,9***	1	58+/−0.82(b)	ND	37.75+/−3.4(a)	ND	42,88*	1	30.2+/−0.28(b)	ND	24.8+/−1.13(a)	ND
*b*	0,01	1	14.12+/−2.02	ND	14.25+/−0.96	ND	256*	1	10.2+/−0.28	ND	7+/−0	ND
*a+a3*	7,63*	1	6.38+/−1.8(a)	ND	10.62+/−2.5(b)	ND	16,79	1	10.6+/−0.85	ND	7.4+/−0.71	ND

Significance of the ANOVA (strain effect) is indicated as follow: * significant at 5%; ** significant at 1%; *** significant at 0.1% (Benjamini-Hochberg correction for multiple testing). df stands for degree of freedom. When ANOVA is significant, Duncan’s class for each strain is noted in bracket. The units are as follow: *K* in cells mL^–1^, *r* in division h^–1^, *lag-phase time* in h, the respiratory rates *JO_2_*, *JO_2_max*, *basal JO_2_*, *JO_2_ATP* in nmol of O_2_ consumption per minute per 10^e6^ cells, *JO_2_ATP/JO_2_* in % JO_2_ due to ATPase, cytochromes *c+c1*, *b* and *a+a3* in pmol/mg dry weight.

### Hybrids having Sc-mtDNA have Higher Respiratory Rate

To go further, the respiratory ability of WU12 hybrids was investigated. Four different respiratory rates were measured: the spontaneous respiratory rate (*JO_2_*, which is oxygen uptake under non-limiting growth conditions), the uncoupled respiratory rate, a proxy for the maximal respiratory rate (*JO_2_max*) achieved by the cells [56], the none phosphorylating respiratory rate (*basal JO_2_*) which is the residual respiratory rate measured when ATPsynthase is inhibited and finally the ATPase respiratory rate coupled to ATP synthesis (*JO_2_ ATP*), which is the respiratory rate due to ATP synthesis functioning. For all respiratory rates, the hybrid having Sc-mtDNA (WU12-8) showed higher respiratory ability (Table 3), with a similar increase of 60% compared to Su-mtDNA (WU12-1). By contrast, the proportion of respiratory rate associated with ATPase functioning was identical (73–76%) in both hybrids. Such a large increase in respiratory rates could be due either to differences in mitochondria number and/or volume, or to variation in intrinsic mitochondrial respiratory abilities. To test these hypotheses, we first run electron microscopy of both hybrids (Figure S1). There was no evident difference in the number of mitochondria, their volume and the number of observed cristae, indicating that both hybrids displayed similar qualitative and quantitative mitochondrial content, independent of mtDNA heredity. We then measured the cellular content in cytochromes *c+c1*, *b* and *a+a3*. WU12-8 Sc-mtDNA showed significant lower content in cytochrome c+c1 cytochromes, as well as significant higher content in cytochrome *a+a3*, in comparison with WU12-1 Su-mtDNA. Interestingly, for cytochrome *a+a3*, a similar trend was observed for DU23 hybrids: DU23-1 Sc-mtDNA harboured higher yet not significant *a+a3* cytochrome content (10.6 pmol/mg dry weight) compared to DU23-4 Su-mtDNA (7.4 pmol/mg dry weight). The cytochrome content of the parental strains revealed that both *S. cerevisiae* parental strains (W1 and D2) had significant higher content in *a+a3* cytochromes (10.0 pmol/mg dry weight) compared to *S. uvarum* strains U2 and U3 (6.2 pmol/mg dry weight), suggesting that variation in *a+a3* cytochrome content might be related to the mitotype. It has been shown in yeast that the respiratory rate is mainly controlled by cytochrome oxidase activity [64] and that during growth on none-fermentable carbon source, cell respiratory rate is directly proportional to cytochromes a+a3 content [65]. Thus, the fact that WU12-8 Sc-mtDNA harboured higher *a+a3* cytochrome content than WU12-8 Su-mtDNA explains the difference observed in respiratory rate between both hybrids during growth.

### Mitotype has no Phenotypic Impact on Fermentation Kinetics and Products

**Figure 5:**
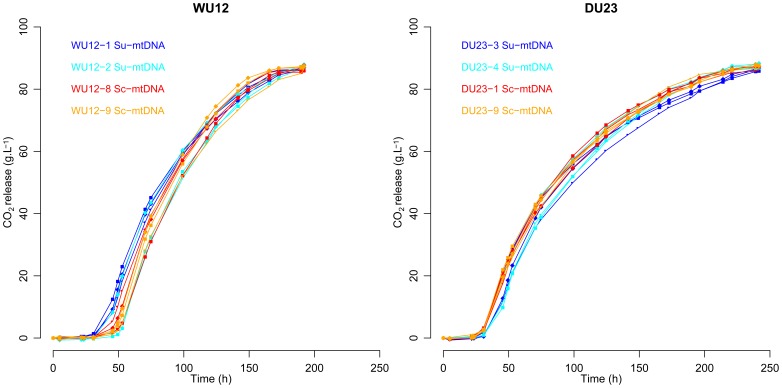
Fermentation kinetics in Sauvignon grape must for WU12 and DU23 interspecific hybrids. Fermentations were performed in 2009 Sauvignon grape must at 18°C in 125 mL bioreactors. CO_2_ release (g.L^−1^) was measured through weight loss. For each strain, the three replicates are represented.

For a long time, mitochondrion was thought to be useless under fermentative conditions, mainly because cells with defective respiration were able to ferment normally [66]. In addition, many genes encoding mitochondrial proteins are repressed under fermentative conditions associating high glucose content and anaerobia [67], [68]. However, several authors suggested that mitochondria may be critical for yeast fermentative performance [44], [69]. Therefore we assessed the possible effect of mitochondrial genotype under fermentative conditions. Alcoholic fermentations were run in grape must, and parameters related to fermentation kinetics (Figure 5) were measured (*lag phase time*, *AF time*, *CO_2_max*, *Vmax*). In addition, at the end of the fermentation, the main products (ethanol, acetic acid, glycerol) were measured, as well as the residual sugar (Table 4). Within each cross, all four strains, whatever the mtDNA genotype, harboured similar fermentative features for all ten fermentative parameters, suggesting that mitochondrial genotype has a negligible effect, if any, in fermentation conditions.

**Table 4:** Results of the ANOVAs: F values and Mean values for fermentation parameters.

	WU12 interspecific cross						DU23 interspecific cross					
	ANOVA		Mean value +/− SD (Duncan’s class)				ANOVA		Mean value +/− SD (Duncan’s class)			
	Fvalue	df	WU12-1Su	WU12-2Su	WU12-8Sc	WU12-9Sc	Fvalue	df	DU23-1Sc	DU23-3Su	DU23-4Su	DU23-9Sc
*Ethanol*	0,49	3	10.97+/−0.08	11.07+/−0.15	10.94+/−0.2	11.03+/−0.12	1,91	3	11,00+/−0.17	10.88+/−0.11	11.15+/−0.17	11.1+/−0.14
*residual* *sugar*	0,21	3	2,00+/−2.77	1.47+/−1.29	0.77+/−0.64	1.67+/−2.37	2,92	3	3.93+/−4.11	6.37+/−3.12	0.53+/−0.42	1.37+/−1.36
*acetic* *acid*	0,81	3	0.05+/−0.03	0.07+/−0.06	0.09+/−0.01	0.09+/−0.02	7,44	3	0.08+/−0.03	0.04+/−0.03	0.05+/−0.04	0.17+/−0.05
*Glycerol*	0,35	3	11.2+/−0.8	10.9+/−0.6	11.2+/−0.6	11.4+/−0.7	0,83	3	9.7+/−0.9	9.3+/−0.8	10.1+/−1.0	10.3+/−0.6
*CO_2_max*	0,63	3	86.78+/−2.04	86.55+/−0.28	87.89+/−1.17	87.25+/−1.04	4,61	3	86.35+/−2.97	84.4+/−0.26	88.89+/−0.75	87.95+/−0.77
*lag-phase*	6,07	3	38.0+/−1.8	42.5+/−4.3	35.9+/−5.8	40.7+/−3.3	1,94	3	26.4+/−2.1	27.6+/−2.2	28.2+/−0.3	24.3+/−2.2
*AF time*	2,12	3	126,0+/−5.0	117.5+/−3.4	110.2+/−5.6	106.2+/−14.6	0,84	3	158,0+/−15.9	150.3+/−0.3	165.5+/−8.6	160.6+/−2.4
*Vmax*	1,39	3	1.26+/−0.02	1.41+/−0.15	1.30+/−0.04	1.41+/−0.09	2,08	3	1.19+/−0.08	1.14+/−0.06	1.05+/−0.03	1.14+/−0.05

Significance of the ANOVA (strain effect) is indicated as follow: * significant at 5%; ** significant at 1%; *** significant at 0.1% (Benjamini-Hochberg correction for multiple testing). df stands for degree of freedom. When ANOVA is significant, Duncan’s class for each strain is noted in bracket. The units are as follow: *ethanol* in percent volume, *residual sugar* in g L^–1^, *acetic acid* in g L^–1^, *glycerol* in g L^–1^, *CO_2_max* in g L^–1^, *lagphase* and *AF time* in h, *Vmax* in g CO_2_ L^–1^ h^–1^.

## Discussion

### Mitochondrial PCR-based Markers: A Useful Tool for Future Research

Previous mitochondrial genotyping in yeast was based mostly on mtDNA restriction patterns, which is time-consuming and unsuitable for phylogenic comparison and recombination studies [70]–[72]. Only one mtDNA PCR-based marker was available (*COX2*) [50], mainly due to the nature of *Saccharomyces* mtDNA showing long AT stretches and short GC clusters [73]–[75], thus limiting the use of PCR approaches. Here, we developed three additional PCR-based markers, two allowing rapid discrimination between Sc-mtDNA and Su-mtDNA (*ATP6* and *COX3*), and two displaying intra-Sc-mtDNA variation (*ATP6* and *VAR1*). Genotyping mtDNA of inter-specific independent hybrids revealed a few events of mtDNA recombination: while for one inter-specific hybrid (WU12) no recombinant mtDNA was found, DU23 hybrid was associated with two stable cases of mtDNA recombination. Although the number of tested hybrids is too low to compare accurately the probability of mtDNA recombination between crosses, these results suggest that mtDNA recombination may vary depending on the parental strains. In any case, our work provides new molecular tools (PCR-based markers) that will be useful to determine the level of mtDNA recombination. Mitochondrial DNA genotyping could now be applied to other hybrids including other *Saccharomyces* inter-specific hybrids but also intra-specific hybrids of *S. cerevisiae*, in order to assess the mtDNA variation according to the genetic backgrounds. In addition, the use of these PCR-based markers may be useful to definitely resolve whether the fixation of one mitotype is stochastic or not in yeast, as different works suggested either random mitochondrial inheritance [41], [42] or non-stochastic one [38], [43].

### Isogenic Yeast Strains Differing Only for mtDNA: An Original Material to Unravel Nucleo-mitochondrial Interactions and Mitochondrial Impact

Previous work addressed the relationships between mtDNA variation and phenotypic traits through the study of reciprocal hybrids in various organisms such as plants [29]–[32], insects [33], birds [34]and fishes [35]. However, in most of these cases, the phenomenon of parental genomic imprinting may be confounded with the effect of mtDNA variability [36]. Here, we exploited the peculiar mtDNA inheritance in yeast to produce hybrids being fully isogenic at the nuclear level, but possessing either Sc-mtDNA or Su-mtDNA. Such a biological material is particularly appropriate for the proper testing of the phenotypic impact of mtDNA polymorphism, in absence of reciprocal parental imprinting. In addition, hybrids differing only for mtDNA could be useful for future investigations regarding nucleo-cytoplasmic interactions. Previous works in yeast revealed nucleo-mitochondrial epistasis in yeast, with phenotypic effect on fitness [76]. Incompatibility between *S. cerevisiae* mitochondria and a nuclear gene of *S. bayanus AEP2* was shown to be responsible for hybrid sterility [77]. Additional ‘incompatibility’ genes were further identified within *Saccharomyces* hybrids of *S. cerevisiae*, *S. bayanus* and *S. paradoxus* [78]. The relationship between cytonuclear incompatibilities and hybrid sterility suggests that this mechanism may be involved in reproductive isolation and subsequently in speciation [79]. Nucleo-mitochondrial interactions (also designed as mitonuclear interactions) may have also played a major role in other evolutionary processes, like in the evolution of sex [80]. Therefore, hybrids differing only for mtDNA may help understanding the role played by cytonuclear interactions in yeast evolution and adaptive ability.

### Mitochondrial DNA Polymorphism has a Phenotypic Impact on Respiration, not on Fermentation

We showed that under fermentative conditions, no phenotypic differences were observed between hybrids having either Sc-mtDNA or Su-mtDNA. It was suggested that mitochondria may play a role in fermentation, in particular because trace amounts of oxygen are necessary for completing fermentation [44], particularly under high sugar concentrations. However, it has been shown that under these conditions, oxygen was not consumed by mitochondria but used for sterol biosynthesis and NADPH-dependent systems localized in microsomal membranes [81]. It should be noted that the fermentative conditions used here were permissive (for oenological conditions), with normal-to-low sugar content (188 g/L). It is possible that under harsher fermentative conditions we may have observed significant differences between hybrids having different mitotypes. Additional analyses under various fermentative environments, from permissive to harsh, will help determining whether mtDNA variation may affect fermentation parameters.

By contrast, under respiratory growth conditions, large differences were associated with the mitotypes. This result is not surprising, knowing that the replacement of the mitochondria of one *Saccharomyces* species by another is usually associated with variation in traits related to respiration [75], [82], [83]. Here, we showed that hybrids having Sc-mtDNA start to grow earlier and faster than their counterparts with Su-mtDNA. The differences in population growth could be related to the respiratory rate (the higher respiratory rate *JO_2_*, the higher the intrinsic growth rate *r* and the lower the lag-phase time). Accordingly, previous work showed that the respiratory rate varied greatly from one strain to another and was related to cell growth in *S. cerevisiae* species [65]. In addition, the differences in respiratory rates between hybrids harbouring either Sc-mtDNA or Su-mtDNA were associated with cytochrome contents variation, particularly with *a+a3* content which appears to be higher for Sc-mtDNA than for Su-mtDNA. It has been shown that electron transfer through cytochrome *a+a3* is a main controlling step in mitochondrial oxidative phosphorylation in yeast [64], [84]. Thus, an increase in cell cytochrome *a+a3* content induce a nearly proportional increase in cell respiration during growth. From a bioprocess point of view, the mtDNA inheritance of interspecific hybrids has to be taken into account for selection. In fact, although some industrial starters used in brewing [85] or winemaking [86] are interspecific hybrids, few studies have investigated the role of mtDNA on their aerobic propagation [87]. The respiratory rate discrepancy observed here between Sc-mtDNA and Su-mtDNA is a key factor that likely affects biomass yield of interspecific hybrids and therefore their subsequent development for industry.

**Figure 6:**
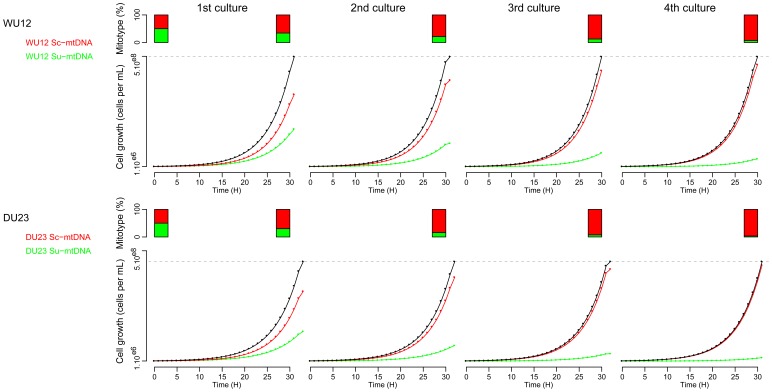
Theoretical evolution of mixed-populations with different mitotypes under respiratory conditions. Modeling population growth was made using the kinetics parameters (maximal population size *K*, intrinsic growth rate *r* and lag-phase) calculated under respiratory conditions (YPEG medium). The initial mixed population contained 10^6^ cells per mL (ratio 1∶1 Sc-mtDNA:Su-mtDNA). When the maximal population size was reached (grey dashed line), the next cycle started with 10^6^ cells per mL. After four cycles, the Sc-mtDNA mitotype represented 92.9% of the total population for WU12, and 96.5% for DU23.

Whatever the molecular mechanisms underlying differences in cytochrome contents and thus in respiratory rates, we demonstrated clearly that mitotypes strongly impact cell growth in yeast, and potentially subsequent fitness. To test this last hypothesis, we predicted the evolution of a yeast population initially composed of 1∶1 ratio of Sc-mtDNA:Su-mtDNA cells. Using the cell growth parameters calculated through logistic fit, we showed that after four recurrent *in silico* cultures with initial population size of 10^e6^ cells *per* mL, the Sc-mtDNA mitotype outcompeted Su-mtDNA mitotype and represented 92.9% of the total population for WU12 and 96.5% for DU23 respectively (Figure 6). Far from the hypothesis that mtDNA variation is neutral, our work shows that mitochondrial polymorphism can have strong impact on fitness components and hence on the evolutionary fate of the yeast populations. From these results, we can hypothesize that the environmental conditions could influence mitochondrial inheritance in interspecific hybrids: under fermentative conditions, hybrids may fix stochastically one or the other mt-DNA, while respiratory environments may increase the probability to fix Sc-mtDNA. The interaction with environments may explain why mitochondrial inheritance was described either as random [41], [42] or non-stochastic [38], [43] in previous works. In any case, our work provides both the biological material and the genetic markers necessary to elucidate the mechanisms of mitochondrial inheritance.

## Supporting Information

Figure S1 Microscopy of WU12 interspecific hybrids harboring either Sc-mtDNA or Su-mtDNA. Several mitochondria per cell are observable (black arrows). The number of mitochondria, their volume, and the number of cristae are similar for both mitotypes.
(TIF)

Table S1 Development of polymorphic mitochondrial markers. ^a^ SGD (http://www.yeastgenome.org); ^b^ GenBank: EU852811.1 [73]; ^c^ range observed for 12 *S. cerevisiae* strains; ^d^ range observed for 4 *S. uvarum* strains; ^e^ RFLP: Restriction Fragment Length polymorphism, LP: Length Polymorphism of amplicon.
(XLSX)

Table S2 mtDNA inheritance for two inter-specific crosses between *S. cerevisiae* x *S. uvarum*. ND: not detected.
(XLSX)
