# Supplementary figures and images for: The Mitochondrial Genome Impacts Respiration but Not Fermentation in Interspecific Saccharomyces Hybrids
Source: PLoS One. 2013 Sep 23;8(9):e75121. doi: 10.1371/journal.pone.0075121 (PMC3781082; doi:10.1371/journal.pone.0075121)

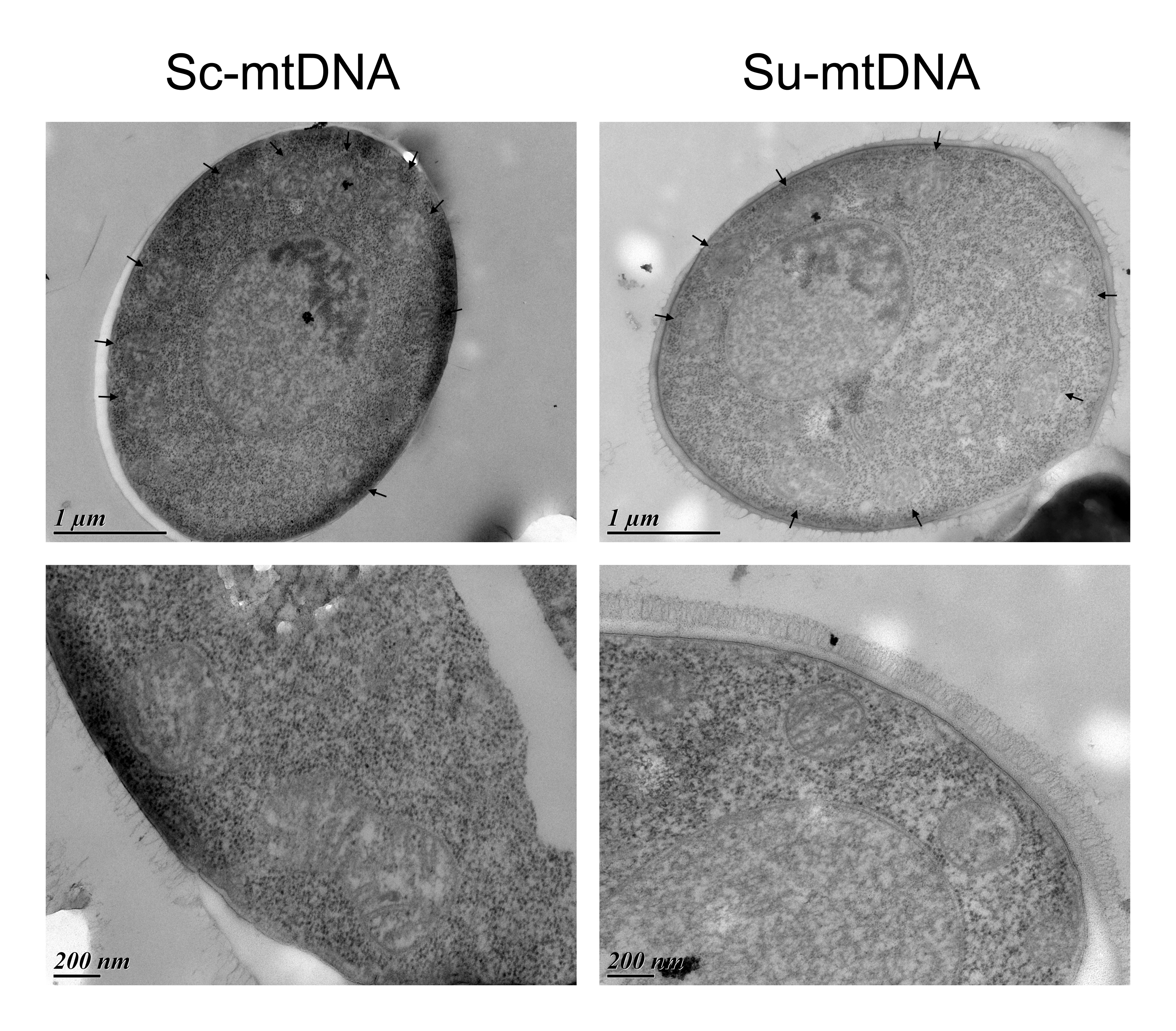

Supplement: Figure S1 — Microscopy of WU12 interspecific hybrids harboring either Sc-mtDNA or Su-mtDNA. Several mitochondria per cell are observable (black arrows). The number of mitochondria, their volume, and the number of cristae are similar for both mitotypes. (TIF) [file pone.0075121.s001.tif]
